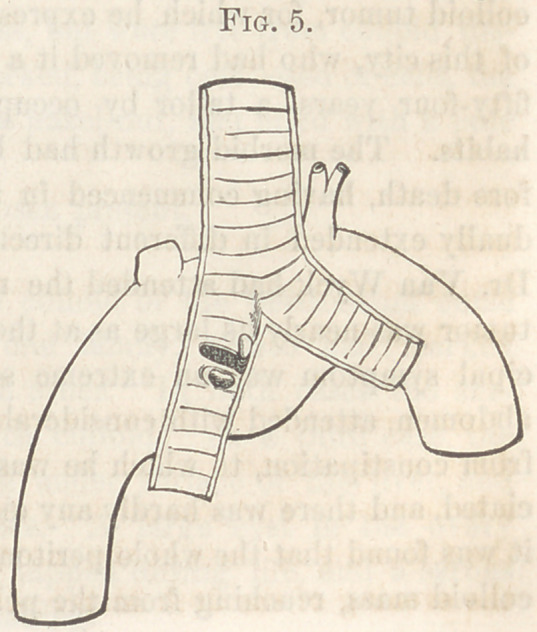# Proceedings of the Pathological Society of Philadelphia

**Published:** 1858-05

**Authors:** 


ART. IV.—*Proceedings of the Pathological Society of Philadelphia.* Reported by the SECRETARY.

WEDNESDAY EVENING, Jan. 27th, 1858.

The President, DR. GROSS, in the chair.

*Cyst Expectorated from the Lung.*—DR. STILLÉ exhibited a cyst about three-fourths of an inch in length, pyriform in shape, and containing a fluid of a yellowish-red color. It was expectorated, not long before death, by a patient of Dr. Gilbert, a man whose main symptoms were great emaciation and cough, and who presented the physical signs of dulness on percussion, and a feeble respiratory murmur on the right side, at the apex of which gurgling also existed. Tubercle was thought to be present, but no post-mortem examination could be obtained, so as to permit the exact condition of the lungs to be stated.

DR. RICHARDSON had seen the cyst shortly after it was coughed up; it was then flat, and not filled with fluid. He considered it an acephalocyst.

*Softening of the Gray Matter of the Cerebral Convolutions in a Child thirteen years of age.*—DR. MITCHELL exhibited a specimen of chronic inflammation and softening of the gray matter of the cerebral convolutions in a child thirteen years of age. The subject from whom this specimen was taken was one of fifteen children, born within a period of sixteen years—the mother having twice had twins.

J. D., when five years of age, was a handsome, intelligent child. At this time he began to see dimly, and at last became entirely blind, from what was believed to be amaurosis. When nearly blind, he began to show a loss of power to fix his attention, and at length, within six years, became completely idiotic. One year ago he was attacked with epileptic fits, which succeeded one another at less and less intervals, until they destroyed his life. Paralysis of the left side, and violent convulsions of the right side, preceded his dissolution. During his idiocy he retained the power of singing, and sang several songs very well, even a few days before he died. He also retained the ability to learn new music, although he mumbled the accompanying words, and evidently attached to them no definite meaning. All other mental effort seemed impossible. His temper was peevish and sullen, and a more pitiable object can scarcely be conceived of.

On post-mortem dissection the following appearances were observed:—Head well developed; body and limbs thin; large ulcer (bed sore) on the sacrum. On opening the head, adhesions existed along the line of the great longitudinal sinus, closely glueing the membranes together; the deposits were of comparatively recent origin. A large amount of serum was found within the membranes, and the brain, compressed and shrunken, did not fill the cranium. With this exception, the upper surface of the organ was healthy, although the membranes were more than usually vascular. Below the middle line of the brain, and principally in the middle and posterior cerebral lobes, were many spots where the surface was a little flattened. On cutting into these, the knife passed through a layer of gray cerebral matter which adhered to the membranes, and then entered a cavity whose walls were ill defined. This cavity was thus bounded on all sides by gray matter, and followed the irregular curves of the convolutions. It contained altered blood discs, brain cells, fragments of nerve tubercles, nuclei, compound granular masses, cholesterin plates, and quantities of granular matter, with occasional fragments of pigment. On careful inspection, the external gray matter of the cerebral convolutions offered, nearly everywhere, some stage of the process whose completed results we have just described. The first stage seemed to be a red line of inflammation just within the external limits of the vesicular neurine. In the later periods of change, this appearance was lost in the complete destruction of tissue which followed. No cause was seen to account for the early amaurosis, and an examination of the retina was denied. The child died, therefore, from chronic peripheral encephalitis with extensive serous effusion.

*Ossification of Arteries in a case of Dry Gangrene.*—DR. MORTON exhibited a specimen of partial ossification of the aorta, right iliac, femoral, and complete obstruction of anterior tibial artery, taken from a patient who died in the Pennsylvania Hospital, of dry gangrene of foot; the disease ran its course in about three months; the vessels of the left side were perfectly healthy.

*Strangulated Hernia.*—DR. MORTON also exhibited a specimen of strangulated hernia, and read the following history of the case:—George Hirst, aged twenty-six, married, was admitted into the Pennsylvania Hospital on January 22, 1858, with symptoms of strangulated hernia. He stated that he had been quite well until the Wednesday previous, when riding on horseback he felt sharp pains in his abdomen; he soon returned home, and after taking laudanum went to bed. The pains continued, and were soon followed by vomiting, which continued all Wednesday; on Thursday he was the same, but with cramps more violent; he then sent for a physician, who did not arrive until Friday morning, when he examined him, and advised his being brought to the hospital, where he arrived about 2 o'clock P.M. On examining the patient I found great tenderness of the abdomen, and a large scrotal hernia on the right side, which was very painful when touched; he stated that his right testicle had always been larger than the left, and that his bowels had not been opened for six days, with the exception of a small passage on Thursday morning. He was placed in a bath, temperature 110°, and an attempt at reduction was made, but failed. Another attempt soon after succeeded better; for the tumor disappeared under the taxis, and seemed to be entirely reduced with the exception of a swelling about the size of an egg, which was no more than a collection of fluid in the sac; since by placing the fingers rather lightly just above the swelling, the fluid could be pressed into the superior portion of the sac. The finger passed up could now feel distinctly the edges of the internal ring; the patient, feeling himself much relieved, was carried into bed, and an injection given. This soon came away, followed shortly afterward by an evacuation of at least a quart and a half of dark-colored fæces. He now felt so much relieved that he fell asleep, and had but occasional vomiting until 10 o'clock P.M., when it again become more frequent.

DR. PEACE saw him at 7 o'clock, P.M., and ordered him a dose of oil, which he vomited, and warm fomentations on the belly; he considered him in a favorable condition, and did not think there was any gut in the ring, but left directions that if symptoms of strangulation became marked, to call a consultation of the surgeons of the Institution early in the morning. At 6 o'clock A.M., the next day, the patient commenced vomiting much more frequently, and soon fecal matter was thrown off; he was soon seen by the surgeon of the house, who, not feeling any obstruction in the ring, thought that the symptoms were caused by an aggravation of the inflammation, or by an internal stricture. At 10 o'clock A. M., the man's skin was cold; belly somewhat tympanitic and tender; scrotum had diminished in size since yesterday. An operation was not thought advisable. He continued to vomit the same way all Saturday, gradually becoming weaker; had voided only about 4℥ of urine since his admission; on Sunday he was delirious; died at 1 o'clock P.M.

Post-mortem Examination.—The intestines were found much inflamed and dark colored, with patches of effused blood here and there, but no lymph, nor any effusion in cavity. On opening the scrotum about two ounces of a thick fluid escaped, of a dark color. The scrotal sac was very large, and seemed as if it had been distended; on running the finger up the cord, a knuckel of intestine was unexpectedly discovered at the internal ring; the intestine was perfectly empty, and quite flabby, and the finger could also be passed around the edges of the ring: the intestine sliding under, and before the finger, gave the impression that no gut existed there; the protuding portion was a piece of the ileum, not far from the cæcum; one side of the gut was attached by bands to the edge of the ring, while the other was quite free, and could be drawn within and without the ring. The probability is that it was an old congenital hernia, capable of partial reduction. The hernia, which was scrotal at the time of his admission, was reduced, with the exception of the small attached portion at the internal ring, which probably had always remained outside, without giving any trouble. The alleviation of his symptoms for the time, and the free evacuation, would be still accounted for; the inflammation continuing, the strangulation finally became more marked.

*Aneurism of Arch of the Aorta.*—Dr. Gross exhibited a specimen of aneurism of the arch of the aorta. The patient, a man aged thirty-seven, had always been in ill health, yet the disease appeared to be only of six months' duration, as far as it had attracted attention. Latterly the marked symptoms were great prominence of the upper portion of the chest; a pulsating tumor; immense difficulty in breathing; an anxious countenance; and great emaciation. About six weeks before death an abscess pointed near the median line, and in three days after it burst and discharged sanious matter; but the immediate cause of death was the opening of the tumor into the pleural cavity.

Autopsy.—The right lung was adherent to the tumor and hepatized; the pleura was thickened, and contained at least a quart and a pint of coagulated blood; the pericardium was adherent to the heart; left lung adherent, and some effusion in left pleural cavity; there was extensive degeneration of the tunics of the tumor; valves of the heart were normal; the orifice of aorta somewhat dilated. The aneurismal pouch was one and a half inches above valves, and contained fibrinous concretions partially organized. The sternum at one place was absorbed, and several ribs had given way; the arteries at the root seemed healthy; innominate artery somewhat dilated; the trachea was not compressed.

Dr. Hewson had seen a case of aneurism of aorta which terminated favorably. The tumor, which could be perceived to pulsate across a room, had eaten its way through the sternum; an abscess, which resulted from caries of the sternum, opened, bone was discharged, and the aneurismal tumor then gradually disappeared. He saw the man two years afterwards, and he was quite well. The case was probably familiar to the members of the Society.

*Erectile Tumor from the Orifice of the Urethra.*—Dr. Packard exhibited a microscopical drawing of a portion of a tumor, removed by him from the orifice of the urethra. The patient, a married woman, aged fifty-two, mother of eleven children, the youngest being now eleven years old, has had hysterical symptoms, headache, debility, &c., for two years, with great pain in urinating. On an examination of the external genital organs, a very florid growth about the size of a lima bean, compressed laterally, and finely lobulated, was found springing from the opening of the urethra, within which its pedicle was attached. It was extremely sensitive, very soft and compressible, but the mucous membrane covering it was tough, so as not to be torn when the tumor was seized with forceps. A ligature was applied to the pedicle, with the effect of cutting directly through it; free bleeding ensued, which was, however, soon checked by dry lint. After soaking in water for hours, the tumor became blanched, and its lobuli less apparent. Examined by reflected light, with a magnifying power of forty diam., it showed an arterial twig ramifying near the surface. In making a section of it, the slice obtained broke down by pressure on the thin glass cover, and the following elements were observed with a power of 360 diameters:—

1. A few squamous epithelial cells.

2. A great many caudate, elongated, granular cells, with large nuclei, sometimes two in one cell, and one or two nucleoli in each nucleus.

3. A great many cells, like those represented by Vogel as seen in "recurring fibroid" tumors.

4. Several wavy masses, somewhat like involuntary muscular fibres, apparently composed of bundles of longitudinal fibrillæ, with here and there a very faint semblance of a nucleus; little change induced by acetic acid.

Dr. S. W. Mitchell, who had examined a portion of the tumor, saw the same elements, also a few nerve tubules. These growths are interesting, from the fact of their frequent occurrence and excessive sensibility, neither of which are accounted for by authors. In the present case there seemed to be no reason to suspect any gonorrhœal or syphilitic affection.

As to their nature, Vogel and Gross place them among the erectile tumors; Rokitansky calls them fibroid, describes them as formed of lax cellular tissue, derived from submucous, and evidently ranks them among polypous formations. The analogy of some cells in this tumor to the confessedly malignant "recurring fibroid," with the known tendency of these sensitive tumors to reappear, together with their alliance with polypus tumors of the nose and antrum, which so often assume a malignant character, would seem, perhaps, to establish something like a chain between simple and malignant growths.

DR. DARRACH examined two erectile tumors some time since, and could find no nerve fibres, although they were exceedingly sensitive.

DR. WOODWARD remarked, in relation to the drawings of the cells of Dr. Packard's case, that the cells resembled those seen in new forming connective tissue. They look like the cells about to multiply by division, seen in new growths of the connective tissue.

*Aneurism of the Innominata.*—DR. HUMPHREYS submitted the following:—John Sweeny, aged thirty-eight, was admitted into the Pennsylvania Hospital Nov. 24th, complaining of a cough which had lasted three or four months, accompanied by a puruloid sputum. He was rapidly losing flesh; suffered from hectic; had occasional attacks of dyspnœa, together with other rational signs of phthisis. The physical signs were not so satisfactory. There was no marked dulness upon percussion anywhere. The respiration was exceedingly roughened throughout the chest, and at the apex of the right lung, bronchial and almost cavernous. The pulse was about natural on the left side, but was wanting on the right. The patient was placed upon the usual treatment for phthisis. About Dec. 20th he began to complain of increasing difficulty in respiration. On the evening of the 23d the dyspnœa increased alarmingly, and about 12 P.M. the patient died suddenly, while sitting upon the close stool.

Autopsy.—The lungs were sparsely scattered with tubercles, a few of which were softened. The bronchi, trachea, and larynx were completely filled with pus, which, during the removal of the lung, was also ejected from the mouth. The heart seemed natural, as did also the aorta, but at the junction of the innominata an aneurism, of about the size of a goose egg, arose, involving the whole of that artery. The whole of the aneurismal sac was filled by dense concentric layers of clot, so as completely to obstruct the flow of blood into the axillary and carotid arteries of the right side. Posteriorly the sac was adherent to the trachea, and at this point, between the trachea and the solidified aneurism, the remains of an abscess were found, capable of holding some two or three ounces of pus, and which had discharged itself through a small opening in the anterior wall of the trachea, thus filling this and the bronchi with pus, and producing suffocation and death. There had been no hæmoptysis, nor any discharge of blood whatever, so that the death of the patient must be entirely attributed to the suffocation produced by the effusion of pus into the air passages, and not directly to the aneurism, as at first might have been supposed.

*Calcareous Deposits in Pleura.*—DR. DARRACH presented a specimen of calcareous deposits in subserous tissue of the pulmonary pleura at the apices of both lungs, with history attached.

DR. DARRACH also exhibited a pneumonic lung:—Mrs. ——, aged thirty-three years, sick ten days, got out of bed some time after retiring, and went down stairs; when she returned to bed she was seized with a chill, and afterwards had great pain in the side, with cough. This occurred on Friday, Jan. 15th. I saw her, for the first time, on Saturday, Jan. 23d.

Symptoms.—Anxious expression of face, purplish lips, panting respiration—44 in a minute; pulse almost imperceptible, intermitting, irregular, and very frequent; excessive pain in the right side.

Physical signs.—Flatness anteriorly on the right side, blowing respiration, prolonged expiration under clavicle; posteriorly very dull, voice hard, no aegophony, no blowing respiration. Left side very resonant, purile respiration, slight mucous rale posteriorly low down.

Treatment.—Antimony, calomel, blister.

Jan. 24th. Breathing freer, pain greatly relieved, pulse unembarrassed—160 beats to the minute, feels stronger. There was not much change after this, excepting that the percussion became clearer, the respiratory murmur increased, and the blowing respiration ceased over the middle anterior region on the right side. The left side remained clear up to my visit on Tuesday, Jan. 26th, with exception of slight dullness very low down posteriorly; hands clammy, anxious. Died at 2½ A.M. on the 27th.

Sectio cadaveris.—Effusion into both sides of chest and into pericardium; left lung had no adhesions, congested, shriveled-looking, probably from compression of the fluid. Right lung adherent anteriorly by a thick yellowish false membrane, slight adhesions laterally and posteriorly, gray hepatization of most of the upper lobe anteriorly; posteriorly the whole lung was very much congested, lower part anteriorly œdematous.

Microscopical examination of the gray hepatization.—Fibrous tissue of lung appeared healthy; the field of the microscope was covered with cells filled with oil, consisting, likely, of both lymph corpuscles and lung cells.

---

WEDNESDAY EVENING, Feb. 10th, 1858.

The President, DR. GROSS, in the chair.

*Cancer of the Great Omentum, of the Smaller Curvature of the Stomach and of the Pylorus.*—DR. DARRACH exhibited a cancer of the great omentum, cancer of the smaller curvature of the stomach and of the pylorus, atrophy of the gall bladder, fatty liver, oily degeneration of muscular tissue of the heart, atheromatous deposits in aorta, on mitral valves, and in splenic artery, peritoneal hypertrophy of the uterus, yellow nodules on ovaries, (probably cancerous,) all obtained from the same patient, a Mrs. ——, aged sixty-six years, born in England, a widow. She was a woman of ruddy complexion, a good liver, indulged in malt liquors; she was of full habit, had been in ill-health for a number of months, and came under the care of Dr. Beesley eight days before death. The symptoms then were frequent vomiting of all she swallowed, (never retaining food for more than three hours, and frequently only for one hour;) pain in region of stomach, which subsided under treatment; abdomen was not tender to touch; laid straight in bed, no evidence of tumour or internal hardening; walked about until three days before her death; insensible for the last twenty-four hours of her life; still vomiting, lost all strength, great nausea, burning pain in stomach, thirst. The treatment consisted in tinct. aconiti, rad. gtt. ij, which relieved the pain, given four times daily; bowels were not opened for eight days previous to death.

Sectio cadaveris.—General appearances: skin clear, ruddy-looking, no rigor mortis. Thorax—heart flabby, fat concealing muscular structure of right ventricle; aorta and pulmonary artery somewhat dilated; muscular tissue of left ventricle pale; slight atheromatous deposits in aorta and on mitral valves. Lungs extremely congested, posteriorly and inferiorly, especially the left, (looked like apoplexy.) Abdomen—peritoneum, over bowels and abdominal walls, much injected; no effusion of lymph Stomach enlarged, a few tubercular-looking deposits along the greater curvature, near the pyloric end, and on the peritoneal coat; lesser curvature puckered up, and closely and firmly attached to under-surface of the liver at the transverse fissure and the fissure for the gall bladder; cancerous deposit at the lesser curvature, near the cardiac end of stomach, and also at pylorus, the latter causing stricture of that orifice; the little finger could just pass through. The deposit at the pylorus was at the upper part, the situation of both deposits being between the muscular and mucous coats; the muscular coat was much thickened, the mucous coat moderately rugous, and covered with small points of ecchymosis. Great omentum was contracted and thickened from its attachment to transverse colon downward; edges thick and rounded; surface covered with nodular tumors, from the size of a pin's head to a small cherry, and of a yellow color; veins of omentum much congested and contorted; fine capillary congestion between the yellow nodules, giving it a very beautiful appearance. Liver; brownish-yellow color—gall bladder about the size of the little finger, with a cavity which would scarcely admit a crow-quill; this atrophy of the gall bladder no doubt was produced by compression, caused by the lymph which was deposited between the liver and stomach, and bound the stomach closely and firmly to its lower surface. Spleen healthy, arteries atheromatous. Kidneys large, pale; supra renal capsules large, softer than normal. Bowels dark-looking, mucous membrane much congested. Mesenteric glands unaffected, as far as examined. Uterus hypertrophied; cavity of cervix filled with a tenacious gelatinous substance; follicles much enlarged, and filled with the same glairy substance. Ovaries full size; each had a small nodule of yellow matter on its surface, of a cancerous nature. Veins of all the organs were filled to a marked degree with black fluid blood; so was the right side of the heart.

A microscopical examination of the deposit in the omentum and stomach exhibited marked cancer cells.

DR. LEVICK inquired whether the splenic artery was not particularly liable to deposits in, and degenerations of its coats. He had seen the splenic and brachial, and the splenic artery and aorta, diseased in cases, in which the other arteries in the body had been perfectly healthy.

DR. GROSS remarked that the splenic artery is more frequently ossified than any other visceral artery. The hepatic and spermatic arteries are seldom or never thus affected.

*Encephaloid of the Right Testicle.*—DR. DARRACH also exhibited an encephaloid cancer of the right testicle, removed from "Mr. ——, aged thirty-five years, single, a farmer, strong, muscular, free from the most remote hereditary taint, of perfectly regular life and habits. To his certain knowledge there has not been cancer in the family for five generations; has noticed, as long as eight years ago, that the right testicle was larger and harder than the left; it has slowly and steadily grown; was painless even on pressure, till within the last few months, when it has had at times a dull aching pain in it, and a feeling of dragging, though a supporter had been worn. From the first the testicle has been equally enlarged, though perhaps the body was rather in advance of the epididymus; surface has been regular, not tuberculated, firm, and latterly almost long in feel over the upper part of the epididymus. Within a few months, a spot on the upper and anterior part of the tumor fluctuated. The cord had always been somewhat enlarged, but was apparently perfectly healthy. On cutting down to it, some fluid escaped from a small unobliterated portion of the tunica vaginalis sac, (which had caused the fluctuation,) leaving the tumor solid." The testicle or tumor was oval, flattened somewhat from side to side, did not cry under the knife; the cut surface marked by fibrous-looking bands, studded with yellow substance, and when cut deeply into, the section presented is of a mottled-red, white, and yellow color. On microscopical examination no trace of the seminal tubes could be found. By pressure, an abundant creamy juice could be obtained, which consisted almost entirely of large characteristic cancer cells. The yellow matter was mostly composed of large cancer nuclei, and cells undergoing oily degeneration, with crystals of cholesterin.

*Enlarged Liver and Spleen.*—DR. STILLÉ exhibited an enlarged spleen and part of a liver, obtained from a post-mortem examination at which he was present. The person, he was informed, a man fifty years of age, and of intemperate habits, was attacked with abdominal pain some months ago, which was followed by dropsical effusion in the extremities and abdomen. Percussion showed the spleen to be enlarged. The urine did not contain albumen, but it was stated by the patient that some time previous to his last illness he had been treated by a physician for diabetes, and recovered. He had never suffered from intermittent fever. The impulse of the heart was augmented. The dropsy increased, and the patient gradually sank.

Post-mortem examination.—The feet and legs were enormously distended. Three gallons of fluid were found in the abdominal cavity. The *liver* was enlarged, and cirrhosed on its under surface. The *spleen* was large and firm; its capsule was dense and opaque, and on it were two white spots the size of a dollar, and of cartilaginous consistence. The *heart* was in a state of dilated hypertrophy; its walls fatty; its valves healthy. One kidney was perfectly healthy, the other was not examined.

DR. FISHER commented on the fact of the diabetes having disappeared. He had observed a case in which the patient had suffered from diabetes, but entirely recovered, and which now exhibited, as in the case just presented, signs of hepatic disease.

DR. DARRACH believed that diabetes occurring at an advanced age, was frequently quite tractable to treatment; sometimes the sugar disappears from the urine to reappear. He spoke of a paper by Jones on the Intermitting Diabetes of the Aged. He had now a case under treatment, in which, for two weeks, no sugar had appeared in the urine.

DR. LEVICK remarked that the liver before them was interesting, in being a case of enlargement, with marked cirrhosis. Dr. L. had seen several cases of marked granular liver, accompanied by enlargement of the organ.

---

WEDNESDAY EVENING, Feb. 24th, 1858.

The President, DR. GROSS, in the chair.

*Atheromatous Deposit in Arteries; Calcareous Deposit at Apex of the Lung; Degeneration of the Pancreas.*—DR. R. P. HARRIS exhibited a specimen of atheroma of the aorta, disease of the pancreas, and calcareous deposit at the apex of the lung, taken from the body of Mr. K., aged fifty-two, of medium height, and sanguine temperament, who was attacked in July, 1857, with what he supposed to be dyspepsia, which was accompanied by nausea and occasional vomiting. He had no fever; did not lose much flesh, and had no symptoms indicatory of inflammatory action. After a sojourn of several weeks at the sea-shore without benefit, he returned to Philadelphia, and placed himself in the hands of his family physician, who subsequently called in the aid of one of his medical brethren in consultation.

At the time he applied for medical aid, he had, together with the nausea before mentioned, hurried respiration, pain in the region of the diaphragm, an inability to inflate the lungs without the production of a short, dry cough, attendant with pain in the epigastrium, an accelerated pulse, and a spasmodic cough. The sounds of the heart were natural, and pressure upon any part of the abdomen gave no immediate pain, unless force enough was used to create pressure upon the diaphragm.

As the disease progressed, the physical signs showed the existence of dropsy in the chest, but nothing special was detected at any time in the lungs themselves, which probably became affected subsequent to the effusion into the cavity of the pleura. The dyspeptic symptoms increased; vomiting became more frequent; effusion into the abdominal cavity occurred, and the legs became anasarcous. The urine, upon examination, was darker colored than natural, (sp. gr. 1012,) and yielded a considerable amount of albumen when boiled and subjected to the action of nitric acid.

Finally the state of the patient became more alarming, from occasional attacks of violent dyspnœa, vomiting, expectoration of bloody sputa, continued difficulty of breathing, drowsiness, prostration, &c. Death took place very gradually, the patient being moribund for two or three days. Respiration became more and more labored; no food was taken; cough ceased (ten days before death;) anasarca diminished slightly; and patient was very drowsy, though not comatose.

From the anasarca, albumen urea, vomiting, and epigastric pain, the disease before death was supposed to be granular kidneys, with perhaps cirrhosis of the liver.

Autopsy, Feb. 16th, thirty hours after death.—Cartilages of ribs ossified, except those of first and second. Pericardium contained about f℥ij. of brownish serum. Heart somewhat enlarged; coronary veins distended, but not ossified. Auricles healthy; secondary pouch in right auricle with very thin walls. Ventricles hypertrophied; left ventricle contained one columna cornea of very large size. Tricuspid valve healthy. Mitral valve slightly thickened with a small cyst upon the opposing surface of the right flap. Both sets of semilunar valves healthy; pulmonary artery the same. Aorta dilated and irregularly studded with atheromatous deposit; primitive, external, and internal iliacs the same. Right lung about half its natural size, strongly adherent to the diaphragm, of a blackish color, covered with deposits of greenish-black disorganized lymph. This lung was entirely hepatized. Left lung hepatized, except at the upper and anterior portion, where it was crepitant, and of a lighter color than the remainder. This lung was much larger than the left. At the upper surface, beneath the clavicle, there was a strong adhesion, in separating which a calcareous deposit was felt, and found upon examination to be imbedded in the substance of the lung, and to extend also into the adhesive band. Pleura contained a considerable amount of dark bloody serum. Liver healthy. Spleen small; marbled light and dark green; lobulated upon the surface; appeared healthy upon incision. Under the microscope, presented no very remarkable appearance; a preponderance of white corpuscles. Right kidney larger than the left; both somewhat congested; cones of the left more pointed than natural. Supra renal capsules healthy. Pancreas smaller than natural; dark colored; almost cartilaginous under the knife; presented fat globules under the microscope.

*Enormous Fibro-Fatty Tumor of the Abdomen.*—DR. GROSS exhibited a portion of a tumor removed, post-mortem, from the abdomen of a man about forty-six years of age. The weight of the entire mass was 74½ lbs. It had been gradually growing for five years, and did not prevent the patient—a superintendent of a foundry in the northern part of the State of New York—from attending to his business, until four weeks before death. The mass, microscopically examined by Dr. Leidy, to whom Dr. Gross was indebted for the specimen, was found to consist of adipose tissue, with fibrous tissue, yellow fibro-elastic tissue, and some bone.

*Tubercle of the Kidney.*—DR. GROSS exhibited a specimen sent to him by Dr. R. J. Levis, of tubercle of the kidney. The patient, Mrs. L., aged twenty-two years, had complained, since a recent pregnancy, of various symptoms not referable to any particular organ, such as debility, slight œdema, neuralgic pains, and nervous irritability. There was slight cough, with profuse mucous expectoration, and the patient finally died, exhausted by a diarrhœa, which attacked her occasionally a short time previous to her death. Her attending physicians, Drs. Jewell and Spencer, state that there had not at any time existed any symptoms of renal disease. The urine, although not minutely examined, presented at no time anything to lead to its particular investigation.

Autopsy.—Body much emaciated, without any œdematous appearance. Right lung tuberculous, with some softened tubercles, and one suppurating cavity. Left lung free from tubercular deposit, and healthy. Heart normal in appearance, but there were about five ounces of fluid in the pericardium. Liver healthy, without any tubercular deposit. Large intestine extensively studded with granular tubercles, and its mucous surface much ulcerated in small patches throughout its whole course. The right kidney was larger than usual, but free from tubercles, and generally healthy. The left kidney was enormously large, and abnormal in its position, being elevated and prominent toward the front of the abdomen. Within its cavity was about a pint of clear serous-looking fluid, without urinous color or odor, distending the pelvis and calices of the organ. The ureter was impervious near its cystic extremity. Masses of tubercular matter varying in size, one nearly an inch in diameter, were scattered through the kidney. The bladder was sound.

*Fracture of Cervical Vertebræ.*—DR. MORTON presented several fractured cervical vertebræ. The patient, a young man, aged nineteen, was admitted into the Pennsylvania Hospital in the month of January, 1858, for an injury to the head and neck, occasioned, as in a case previously shown, (see *Proceedings*, Nov. 25th, 1857,) by falling into the hold of a vessel and striking the back part of his head and neck. On admission he had symptoms of concussion, great pain in the back of neck, priapism, entire loss of sensation and motion from his arms down. His neck had the appearance of being dislocated; it seemed shortened, and the head thrown forward. His water was drawn from him for the first thirty-six hours, after which his fæces and urine passed from him involuntarily until his death, which took place on the tenth day. He remained perfectly conscious until a short time before his death.

On post-mortem examination, the bridge which connects the spinous and oblique processes of the fourth cervical vertebra was broken through; its body was also split. The bridge of the fifth was broken in the same way as the fourth, and its body split through and through. The right side of the vertebra was dislocated anteriorly, the oblique process had slipped forward and over that of the sixth. The body of the sixth was split entirely through and through. The spinal cord was pressed upon in several places, and large clots of blood could be drawn from the canal.

DR. HEWSON drew attention to the fact of the patient having lived nearly eleven days after the occurrence of the fracture. He had seen a patient who received an injury to the back, which caused the fracture of his fourth, fifth, and sixth dorsal vertebræ. This patient lived three months after the accident. There was an angular projection and displacement at the seat of fracture The fifth or sixth dorsal vertebræ were found to be pressed downward, obliterating the spinal canal. A bony growth had been thrown around the seat of fracture.

*Suppuration and Enlargement of the Kidney.*—DR. HALL called the attention of the Society to a specimen of abscess of the kidney.

The patient was a thin, ill-looking, anemic young woman, twenty years of age, a shop-girl by occupation. She was admitted, March 17, 1857, into the Pennsylvania Hospital, under the care of Dr. Pepper, for "vaginitis." She stated that she had been sick eight weeks with "pain in her privates," and a scalding when she made water. A fall from a chair was the cause assigned by her for the discharge and pain. The discharge from the vagina, with concomitant circumstances, was such as to warrant at least a *suspicion* of its specific origin. The girl herself, who was single, was either by nature exceedingly slow of intellect, or else by design kept to herself any information that she might have to communicate.

She never spoke unless spoken to, and then replied briefly and petulantly, which may be mentioned to account for the meagre history of the case. She was placed upon a tonic and specific treatment, with a nutritious diet.

March 27th. Fever, hot skin, flushed cheeks, accelerated pulse passed off in three days, under appropriate treatment.

April 10th. The vaginal discharge had ceased. She was terribly emaciated. Ol. morrhuæ was ordered, in hopes of fattening her, and used for three months without benefit, then discontinued from the nausea caused by it. Auscultation and percussion revealed no disease of the lungs. The urine, on standing a little while, let fall a thick, creamy, yellowish opaque deposit to the bottom of the vial. This deposit, under the microscope, presented the appearance of pus granules, and the addition of liquor potassæ produced its peculiar action. The amount of urine passed in the day was normal; the specific gravity also. No albumen could be discovered in it from time to time; no hæmaturia while under observation. To remove all doubt as to the source of the pus, a specimen of urine, drawn by catheter from the bladder, was subjected to examination. This produced the same results. The pus was thought to come from the kidney. A circumscribed tumor, dull on percussion, in the left hypochondriac space, could be felt on relaxing the muscles of the thigh and abdomen. Posteriorly, the tumor presented a slight bulging externally. It was painful upon firm pressure being made on it. As symptoms of exhaustion began to show themselves, milk-punch and wine were added to the treatment.

July 16th. The patient at this time presented typhoid symptoms which, however, seemed readily amenable to treatment, temporarily at least. The tumor remained as at last notice, neither diminishing nor showing any signs of marked increase.

July 24th. So far the patient has been remarkably taciturn, but fretful when moved in bed. Now, she seems more indifferent to matters around her. Sleeps most of the day and night. Has involuntary discharges from the bowels and bladder. She now most plainly begins to sink. There were frequent spells of vomiting, the matter ejected being of a glairy, brownish nature, at times nearly of a chocolate color. The patient continued to sink, and died August 17th.

Autopsy ten hours after death.—Body more emaciated than Dr. Hall had ever beheld before—the thighs could be spanned by the thumb and forefinger. Cadaveric rigidity not well marked. Head—there was from f℥iv. to f℥v. of serum in the sac of the membranes, and in the lateral ventricles. There were no changes in the brain substance. Thorax, heart, and great vessels perfectly healthy. Lungs perfectly healthy, but exceedingly pallid, floating like a cork in water. Liver merely pallid. Spleen normal, if anything rather small. Right kidney smaller than usual, somewhat congested; right ureter healthy. Left kidney very much enlarged, looking like a large oblong tumor, as it laid in the left lumbar region; it was raised with difficulty from its bed, being bound down firmly to the surrounding textures. The capsule of the kidney was firm and dense, cutting like cartilage, produced, it was thought, by chronic inflammation. In removing the organ, an accidental cut was made across the ureter, close to where it emerges from the kidney; a few drops of curdy pus protruded from the orifice. The kidney measured seven and a half inches in length, and weighed fifteen ounces—nearly three times its natural size and weight. On making a section of the organ, it at first appeared to be one mass of pus; but on washing away some of this, its structure did not appear to be entirely destroyed. The cortical portion was that most affected; in it were several abscesses of different size, the largest probably as big as a walnut. The cavities of these abscesses were ragged and sloughy; the tissue a little beyond and around them was condensed and hardened. The tubular portion of the kidney appeared to be comparatively little involved. The left ureter was enlarged to twice the size of its fellow, and its coats were thickened; it was pervious throughout its course. The urinary bladder presented spots of injection on its mucous membrane. Uterus presented nothing peculiar. The left ovary contained a large cyst. Vagina—traces of inflammation, deep-red injection, especially near the os uteri; there was no hymen.

This case presents many points of interest. In the first place, the possibility of the communication of the inflammation from the vagina to the bladder, thence by the ureter to the kidney, thus producing the mischief that resulted in death. Secondly. The absence of albumen in the urine, as examined from time to time; the normal specific gravity always exhibited; the natural quantity of urine passed in the day. Thirdly. The absence of œdema, or dropsy of the lower extremities. Also the absence of constitutional irritation in proportion to the amount of disease present. Also, the form and character of pain were not such as the gravity of the lesion would seem to warrant.

A point that should further not be overlooked, was the small size of the right kidney, considering the double service that it had to perform, the left kidney being totally useless as an excreting organ. That it, the right kidney, was not hypertrophied is a matter of great surprise.

DR. LEVICK stated that he remembered the urine in the case just reported by Dr. Hall to have contained pus. Toward the close of her illness, a swelling was observable over the region of the kidney. The specimen he had believed, when he examined it, to have been a scrofulous or tuberculous kidney.

DR. GROSS thought the kidney had more the appearance of encephaloid, than either of suppurative nephritis or of tubercle. Simple suppuration would not present the organ so much increased; for tubercle, also, the kidney is too voluminous, and the absence of tubercle in other organs is against its being such. Dr. Gross then inquired if any members of the Society had observed tubercle of the kidney unassociated with that of the lung. He had seen a case of this kind, in which the left kidney alone was the seat of the disease. There was a psoas abscess, and five ulcers were found in the urinary bladder.

DR. LEVICK also mentioned a case affecting only the left kidney.

DR. DARRACH had had two cases of tubercle of the kidney come under his notice, in one of which no tubercle at all existed in any other organ of the body, while in the other there were some scattered miliary tubercles in the lung. In the first case, one kidney only was found to be affected. The urine contained a heavy deposit, which, when examined with the microscope, showed corpuscles like those of tubercle, and having the same reaction with acetic acid. In the second case, there was extreme diuresis, and no evidence of tubercle in the urine.

*Dilatation of the Heart; Insufficiency of the Tricuspid Valve.*—DR. PACKARD made the following remarks on the pathological appearance of a case of extensive dilatation of the heart, accompanying disease of the tricuspid valve:—

A boy, aged sixteen, had been troubled with a slight cough for several years; according to the account given by his friends, he never had suffered from rheumatism; he had, however, always been somewhat weakly. Owing to circumstances, I had only the opportunity of making an autopsy, without having examined him during life; and these notes are presented simply because the lesions described therein have seemed to me difficult to explain.

Autopsy twenty-six hours after death.—Rigor mortis well pronounced. Body small and ill-developed, and much emaciated; some œdema of the extremities. On cutting through the sterno-costal cartilages, a considerable quantity of clear, yellow, somewhat ropy serum escaped. The two layers of pericardium were almost universally adherent, the exception being over a portion of the right ventricle and auricle, and over the commencement of the pulmonary artery; here a good deal of clear, yellow, jelly-like lymph had been thrown out, evidently very recently, so as at once to make and to occupy an interspace; I presume the yellow liquid, before alluded to, was the thinner portion of this lymph. The heart was about eight inches in length; its walls did not seem much thickened, except those of the right ventricle, which were so to a considerable degree. All four of the chambers were of increased capacity, especially the left auricle, which would have contained about six fluidounces; just above its auricular appendage was a secondary pouch formed in the anterior wall of the sinus venosus, like a sacculated aneurismal dilatation; it was about one inch in length, formed mainly of endocardium, and would readily admit the point of the fore-finger. On the right side, the auriculo-ventricular valve had but two flaps, one of which was so closely adherent to the septum ventriculorum as scarcely to admit of the insertion of the finger or knife-handle beneath it; the closure of the orifice was decidedly insufficient. The corresponding valve on the left side was somewhat thickened and insufficient; its chordæ tendineæ and columnæ carneæ were of more than normal volume. Both sets of semilunar valves were healthy, as were also the great vessels, as far as traced. No signs of endocarditis presented themselves. The lungs were healthy; but there were calcareous deposits in some of the bronchial glands. Liver, kidneys, supra-renal capsules, and spleen normal.

The pericarditis was here evidently the latest lesion; but I am completely at fault to explain the rationale or the order of sequence of the other abnormal conditions. On the left side, where the insufficiency of the auriculo-ventricular valve was less marked than on the right, and where consequently there must have been less regurgitation, the auricle was hypertrophied to a far greater degree. The adherent flap of the right auriculo-ventricular valve I presume to have been rendered so by some former attack of endocarditis; but such adhesions, I believe, are very rare; nor are cases of non-rheumatic peri or endocarditis frequently met with.

---

WEDNESDAY EVENING, March 10th, 1858.

The President, DR. GROSS, in the chair.

*Malformation of the Heart, Communication between the two Ventricles, and between the Auricles; Cyanosis.*—DR. DARRACH exhibited a malformed heart, taken from an infant, aged eight weeks, of German parents: child was blue from birth; cried with much strength; tongue blue.

Sectio cadaveris twenty hours after death.—Head not examined. Thorax —lungs somewhat collapsed, no adhesions, of a florid hue, easily inflated with air from a pipe introduced into the trachea. Heart—small amount of fluid in pericardial sac, pericardium healthy; heart of a modena hue, coronary arteries empty, veins full; organ of a roundish form, looking, as was remarked, like a fish's heart. The aorta was situated to the right side, and much larger than normal. The pulmonary artery, instead of occupying its usual position in front of the aorta, was situated at the left side of it, and about one-fifth or less of its usual size. On opening the heart, the aorta was found to arise from the right ventricle, and an opening, *about large enough to admit a large quill*, semilunar in form, existed at the upper portion of the septum ventriculorum. There was an entire obliteration of the pulmonary artery at its junction with the heart; but from this point upward it was pervious to the lungs, communicating with the aorta through the open ductus arteriosus. The walls of the two ventricles were nearly of the same thickness, the left a little thicker than the right; the auricular ventricular valves were healthy; the semilunar valves were also normal. There was an entire want of the septum auriculorum. Abdomen—the viscera were all healthy. There was an anomaly in the vena cava, which should be noticed It divided soon after leaving its fissure in the liver, into the two iliacs; the renal veins thus being given off by the iliacs.

At first glance there appeared in this case nothing but disorder, or an entire pathological condition; but, on more careful and thoughtful examination, we can perceive, as in all disease, the workings of a "protective" as well as a "destructive principle." The grand fault of nature in this case was no doubt the closure of the pulmonary artery, thus preventing the exit of the blood from the right ventricle; and unless some substitute for the pulmonary artery could be made, all circulation must have ceased. This was accomplished by shifting the aorta to the right side; and that the left ventricle might not be neglected, an opening was made, acting as a communication between the two ventricles; and so that the blood could reach the lungs, the ductus arteriosus was left open. We had, then, functionally, but one ventricle, and on this we are able to explain the want of septum of the auricle; for, having but one ventricle, we needed but one auricle, making thus a batrachian heart.

*Ovarian Cyst in an Infant eight weeks old.*—On removing the uterus of the above infant, a cyst was found, developed from the left ovary, about the size of a small marble.

DR. KEATING inquired whether there had been atelectasis at birth.

DR. DARRACH.—None; the child screamed lustily as soon as extruded.

DR. DARRACH exhibited another specimen of open foramen ovale. The heart was obtained from an adult man.

DR. KEATING spoke of several cases of open foramen ovale which had come under his notice, in which the persons had not suffered any inconvenience. In one case the sixtieth year was attained without there being any cardiac, pulmonic, or other disturbance. Dr. Keating, in reviewing the opinion held with regard to the open foramen ovale being the essential cause of cyanosis, expressed his dissent from this view. Obstruction of the pulmonary artery he believed to be a more active cause. But he had seen want of expansion of the lung at birth, unconnected with either of the above-mentioned states, give rise to the blue appearance, which had disappeared under appropriate treatment.

DR. DARRACH inclined to the belief that obstruction of the pulmonary artery was mainly concerned in the production of cyanosis. He alluded to the statement of some authors, that the foramen ovale was kept open by nature, on account of the obliterated pulmonary artery, so as to act as a safety-valve.

DR. MITCHELL suggested as the reason why an open foramen ovale might exist during life without occasioning any disturbance, that in reality no venous blood would mix or circulate with the arterial, owing to the left auricle being stronger than the right. Its more powerful muscular fibres would send arterial blood to the right side of the heart, which, of course, going to the lung, would come back to the left auricle; but no venous blood, owing to the feebler contraction of the right auricle, could get into the left side of the heart, and from there into the general circulation.

DR. DARRACH thought Dr. Mitchell's theory to explain the absence of cyanosis, where an open foramen ovale existed, was open to objections, and mainly on account of its anatomy. It had appeared to him in dissection of the heart, that no difference between the auricles occurred. If any, the right was the most powerful.

*Encephaloid of the Kidney.*—DR. DARRACH next exhibited a specimen of fungoid cancer of the kidney, with accompanying microscopic drawings. The history was given as follows:—Mr. B., aged fifty-three, German, married; large; generally enjoyed good health; steady drinker of brandy. The attending physician stated that four years ago he fell heavily upon his back, and from that time had felt almost constant pain in the right lumbar region. About a year before his death he passed a large amount of blood by the penis, and had had several hemorrhages since, up to his death. I examined his urine several times, a short time before death, but found nothing abnormal, with the exception of blood, and bile coloring matter. The urea was in normal quantity. The patient suffered very much from the pain in the lumbar region, during the latter part of his sickness. A marked amount of effusion accumulated in the abdomen at one time, which accumulation, together with a yellow hue of skin, and bilious urine, led to the diagnosis of cirrhosis of the liver. A few days before death, our attention was attracted to the right lumbar region, from his complaining of pains, when a nodular lump was felt, and on percussion marked flatness was perceived. At first, the liver was thought to be enlarged, and extending into the lumbar region; but as the fingers could be passed between the lower edge of the liver, and this nodular mass, by a process of exclusion, and taking into consideration the history of the case, and the bloody urine, we came to the conclusion that he had a cancerous kidney as well as cirrhosis of the liver.

Sectio cadaveris.—The head and chest were not examined.

Abdomen—there was considerable effusion in the peritoneal sac. The organs all appeared healthy, with the exception of the liver and the kidneys. The liver was enlarged and granulated, especially on the under surface, and of a brownish-yellow hue. The right kidney was about three times as large as normal, very much misshapen and nodulated, the size of the nodules varying from that of a small to a large walnut, some of them of a dark-black color, the rest of them, with the other portions of the organ, being of a light-yellow and red hue; the surface was much marked by brightly injected vessels, many of them having a stellate arrangement. On opening the kidney the nodules were found to be cysts; the dark-black ones filled with coagulated blood, while the others contained matter having a mottled-white, yellow, and red appearance, not unlike a well-marked nutmeg liver; this, on examination with the microscope, was shown to consist of oil globules in abundance, granular matter, and cells, which had the appearance of ordinary kidney-cells, many of them entirely filled with oil globules, others partially so, and exhibiting their nuclei. The left kidney was much hypertrophied, but to the eye, and under microscopical examination, there appeared nothing abnormal.

DR. WOODWARD showed a heart-clot, taken from the right side of the heart. It had probably formed before death, and consisted of white masses of unorganized fibrillated fibrine. The man—a sufferer from valvular disease of the heart—died suddenly. The aortic valves were much diseased. Dr. Woodward brought forward this specimen to disprove the opinion that heart-clots can only be formed during a protracted agony. This had formed in a case in which death was not protracted.

DR. FORBES, who had examined the heart from which this clot was taken, when recent, stated that in addition to the extreme ossification of the aortic valves, the valves on the right side of the heart had been diseased. Those of the tricuspid were thickened, those of the pulmonary artery rigid from a deposit. The left auricle was extremely thin and dilated.

*Hepatic Abscess discharging through the Lung.*—DR. MOREHOUSE called the attention of the Society to a large hepatic abscess. The patient from whom the specimen was taken suffered with repeated attacks of dysentery from early in September last until late in December, the date when disease of the liver became manifest. The attacks were frequent, almost running one into another, but so mild as scarcely at any time to confine him to the house. On the 25th of December he felt chilliness, loss of appetite, with slight fever at night, but attributed them to a cold, and was content to take such measures for their relief as his own judgment dictated. He continued to go out, in spite of his increased sickness, until the 1st of January, when he was suddenly bent double with pain in the right side, and in that condition brought home cold and prostrate from the intensity of his suffering. This pain continued, with slight remissions, until he died, on the 27th of February. During the progress of the abscess there was occasional nausea, and in the latter weeks, pain in the right shoulder. The dysentery disappeared entirely, as also the tenderness of the abdomen, except over the liver. The right side enlarged, with dulness on percussion over the epigastric and right iliac regions. The sixth and seventh ribs were widely separated, and in the intercostal space obscure fluctuation could be detected. Such was the condition until a few days before his death, when he was seized with a suffocative paroxysm, followed by the expectoration of a pint or more of "dirty red, puriform matter," considered by some as pathognomonic of abscess perforating the lung. At the end of two days the purulent expectoration had nearly ceased, although a constant hacking cough, with oppressed breathing, still remained. The enlargement of the side was not apparently reduced by the discharge, but the pain was very decidedly relieved. Five days after the opening of the abscess he died.

Post-mortem examination.—The body was emaciated, but not in the least discolored; the right side was very prominent, and fluctuation was evident between the sixth and seventh ribs and along their lower border as far forward as the epigastrium. On opening the abdomen, the liver was found attached firmly to its walls, from the ensiform cartilage to the lower border of the iliac region. The right lobe, in which alone disease existed, was converted into an immense cyst, from which was taken six pints of pus, similar in color and consistence to that expectorated a few days previous. There were, however, shreds of tissue, and a layer of white pus adhering to the disintegrating surface of the liver, which contrasted strongly with the prevailing color of the mass. The diaphragm formed the upper wall of the abscess, and at one point presented the appearance of having been perforated, but was now entirely closed. The left lobe was natural in size and structure. The gall-bladder contained about half a gill of thick bile, which was so inspissated and hardened at the neck as to be readily discerned from the outside. In the large intestine were three cicatrices of ulcers, two in the cæcum and one in the descending colon, probably the most recent. The lower lobe of the right lung was infiltrated with colored pus, and adherent by its base to the diaphragm to an extent not exceeding two inches in diameter. In the apex of each lung were tubercles, some of them undergoing calcareous transformation. Throughout this case there was not the slightest jaundice, nor could bile be detected in the urine by its color, or by the reaction of sulphuric acid. This is most remarkable, considering the extensive disorganization of the liver, and the consequent suppression of two-thirds, at least, of its secreting power. It cannot be supposed that the remaining left lobe, which was not enlarged in mass, and did not indicate under the microscope an increased number of secreting cells, could assume the functions of the entire organ. Moreover, this conclusion is disproved by the occurrence of jaundice in cases of obstruction of the right hepatic duct, when the same amount of secretive tissue, as in this specimen, remains unimpeded in function. The evidence of this case, therefore, would seem to conflict with the theory that bile exists as such in the blood, and that deficiency of its elimination is the proximate cause of jaundice. Most of the cases of hepatic abscess of the right lobe that have been recorded, corroborate this view. Out of those given by Annesley, in his work entitled *Researches on the Diseases of India, and of Warm Climates*, among which are several where the function of the liver was more completely destroyed than in the case above cited, there is not one mentioned where jaundice occurs as a symptom. Andral, in his *Medical Clinic*, records one case with jaundice, but then the abscess was on the lower surface of the right lobe, and very liable to compress the hepatic ducts, which converge toward that part of the liver.

DR. GROSS did not feel quite satisfied that the matter expectorated came from the hepatic abscess. No seat of perforation was visible, and the abundant secretion might have come from the inflamed bronchial tubes and parenchymatous structure of lung.

DR. MITCHELL suggested that inflammation might have so closed up the opening into the lung as to prevent this from being discovered.

*Fibrous Tumor of the Uterus removed during Labor.*—DR. KEATING exhibited a specimen of fibrous tumor of the uterus, and accompanied it by these remarks:—

I was called to attend Mrs. W., of Vine Street, in her fourth confinement, at 3 A.M. on Monday the 14th of February.

Mrs. W. enjoyed excellent health during the whole period of gestation; but her enormous size had caused her to believe that she was pregnant with twins. About eighteen months previous I had delivered Mrs. W. of a still-born female child, presenting in the second position of the breech. The child seemed to have died some hours before the access of labor; and as the delivery was rapid, and in every respect natural, I was at a loss to account for its death, all attempts at resuscitation proving useless.

Upon entering the room on the morning of the day mentioned, I was informed by the nurse that the membranes had ruptured, and that a large amount of liquor amnii had been discharged. Upon making a vaginal examination, my finger came in contact with a large fleshy mass, which at first I considered a placenta prævia; but as the hemorrhage was inconsiderable, and a second examination did not confirm my suspicions, and especially as the os uteri was not dilated beyond the circumference of a half dollar, I concluded to await further dilatations of the os uteri previous to deciding as to the presentation. The labor-pains were slight and irregular, producing but little effect; the os remained firm and undilatable, but a portion of it seemed to be included within the presenting body, so that I could not with certainty define the posterior lip of the os uteri.

At 6 A.M., finding that the labor seemed obstructed, and that the contractions of the fundus uteri had but little effect upon the os, and not being able as yet to ascertain the presentation, I resolved on dilating with my finger the anterior lip of the os uteri. I succeeded partially, but met with great difficulty, from the size and resistance of the presenting body, which seemed completely to fill up the opening of the uterus. My examination, however, was sufficient to convince me that I had not to deal with a case of placenta prævia. From this I surmised that the body in question might be one of those cystic tumors of the nates, two cases of which have been presented to the Society by my friend Dr. Keller.

Finding at 6½ A.M. that the labor made no progress, and being still uncertain as to the nature of the case, I requested a consultation with Dr. Keller. In his first examination, he was also inclined to adopt the idea of a placenta prævia, but repeated examinations, attended with some violent expulsive efforts on the part of the patient, causing a sudden and partial protrusion of the presenting body, convinced us that it was a large fibrous tumor. Subsequent labor-pains, and further dilatations of the os uteri, enabled us to detect the right shoulder of a fœtus resting upon the tumor, with the umbilical cord prolapsed and pulsating. Fully satisfied as to the nature of the case, and convinced that no delivery of the fœtus could take place without the previous removal of the tumor, we requested the assistance of Dr. Addinell Hewson, who, after careful examination, fully coinciding in our views, immediately proceeded to enucleate the tumor, preferring this method of removal to that of excision, as a matter of prudence in case of threatening hemorrhage. The pedicle of this tumor measured at least three inches in diameter, and consisted of a dense fibro-areolar membrane of the same character as that which invested the whole tumor. It was this dense and unyielding character which caused me at first to suspect that the presenting body was not placental. The enucleation was rapidly and successfully performed by Dr. Hewson, and was attended with little pain and slight hemorrhage. The tumor, upon extraction, weighed $2\frac{3}{4}$ pounds, and measured twenty inches in circumference. It was fibroid in its character, and was firmly attached to the posterior portion of the os and cervix uteri, entirely obliterating that lip of the os; its pedicle extended half an inch within the os internum, and after the removal of the tumor a sort of auricle was left, which might be considered as the undeveloped portion of the cervix uteri, the anterior portion having obeyed the usual law of pregnancy, and entirely disappeared.

From 8 to 10 A.M., the uterus remained in complete inertia, and the patient seemed exhausted. The anterior lip of the os had again contracted, and it was impossible to resort to version. A drachm of powdered ergot was administered in divided doses, and frictions applied externally over the abdomen. Subsequently we commenced dilating the os with our fingers, and succeeded in arousing some uterine contractions; version by the head was then accomplished, and the forceps applied to the head of the child as soon as it engaged in the pelvis, inasmuch as the patient was in a complete state of prostration, and made no expulsive efforts. Notwithstanding all our efforts for a rapid delivery, and our subsequent attempts by means of Marshall Hall's ready method of resuscitation, all proved useless; the child was dead. It weighed $9\frac{1}{2}$ pounds.

An alarming flooding supervened after the birth of the child, which was checked by an artificial delivery of the placenta, the introduction of ice into the vagina, and by stimulating the uterus to contraction by the introduction of the hand. The patient remained in a favorable condition until the following Wednesday morning, when violent symptoms of puerperal fever set in, of a phlebitic character; in spite of all our exertions to save her, she died on the following Monday night. No post-mortem examination was allowed.

The peculiarity of this case consists in the existence and attachment of such a tumor to the os and cervix uteri without producing any morbid symptoms, and without provoking abortion in the early stages of pregnancy. Her menstrual periods had been regular as to time and as to quantity; she had never felt any inconvenience from the presence of this tumor, but I strongly suspect that its existence, though not as much developed, had caused the death of the fœtus in her previous confinement.

It is evident that such cases as these give the *coup de grâce* to the theory which makes the os and cervix uteri the guardian of pregnancy, and which claims an antagonism as existing between the fundus and cervix from the earliest months of pregnancy. Were such views correct, it is evident that the os and cervix, with such a tumor attached to their posterior parietes, would be in no condition to struggle against the supposed continued contractions of the fundus. Under such odds the early defeat of the enfeebled cervix must have inevitably resulted. Some astonishment has been manifested in reference to conception taking place under such circumstances; but a moment's consideration would suggest the response that the anormally patulous condition of the os and cervix uteri would rather favor than oppose such a result.

*Report of Committee appointed to examine the Kidney presented by Dr. Hall.*—The microscope exhibited what were supposed to be epithelial cells, and a large number of corpuscles, which, from their size, shape, appearance and reaction with acetic acid, resembled tubercle. Some cells in regard to size and form might be considered as pus-cells; but the reaction of acetic acid did not develope any nuclei. No cells were found which in the slightest degree resembled cancer-cells. The Committee, from a careful examination of the specimen, are disposed to believe that it is tubercular.

Signed DRS. DARRACH, PACKARD, MORTON.

---

WEDNESDAY EVENING, March 24th, 1858.

The President, DR. GROSS, in the chair.

*Deposit in Spleen, and Fatty Degeneration of Supra-renal Capsule.*—DR. HEWSON exhibited a spleen filled with deposits, a supra-renal capsule in a state of degeneration, and a gall-stone, removed at the autopsy of R. S., aged seventy-seven years, a patient of Dr. ——. Mrs. S. had been under Dr. ——'s care about seven weeks previous to her death. When first called to her, Dr. —— found her suffering with great dyspnœa at night, and complaining of pain in the left hypochondriac region. She was much icterode, and her stomach was excessively irritable. There were no signs of cardiac disease, but in the right hypochondriac region he felt some nodular masses, and supposed them at this time to be cancerous masses in the liver; and in this opinion he was confirmed by Dr. ——, who saw her shortly after with the physician first called to the case. Tongue had been furred, dry, and cracked across; thirst was never great until a week before her death, when she showed great avidity for everything to drink. There was never any great heat of skin; pulse generally about 85. Her bowels were costive, defective in bile, and she always obtained considerable relief from their evacuation.

Autopsy fifty-six hours after death.—Body not much emaciated, legs œdematous. Abdomen—liver small; its envelope in places covered with fat; organ in a state of decided fatty degeneration; gall-bladder distended, with thin fluid bile, and contained a large gall-stone, (82 grs. weight,) beautifully bright and glistening, of an orange hue, and quite translucent. Stomach not at all distended, but presenting, at its pyloric orifice, well-marked signs of long-continued gastritis. Its mucous membrane was mottled, with patches of deep dark-red, exceedingly soft and pulpy, and with spaces of beautiful arborescence. The mucous membrane of the duodenum was in the same state. There were no obstructions in the ductus choledochus or hepatic duct; pancreas was perfectly healthy. Spleen small, natural in appearance, but had on its surface two white patches of probably fibroid deposit, one the size of a pea, the other over half an inch, and extending fully that depth into the substance of the gland. Right kidney perfectly healthy; left kidney had a cyst on its surface, near the upper end of the gland; the supra-renal capsule was in a state of fatty degeneration. Bladder much distended, but healthy. Uterus small, and smooth on its external surface; was studded with melanotic deposit on its mucous surface, and there was some ulceration at the os tincæ. Thorax; heart—no great quantity of fluid in the pericardium; surface of heart covered, to some extent, with fat, organ of full size, and remarkably healthy in every respect, no paleness of structure or atheromatous deposit; aorta had a number of such deposits along its whole course. Lungs were of a dark-blue color, adherent in a number of places by old pleuretic adhesions; no signs of tubercle or congestion; both lungs were rather emphysematous throughout.

*Atheroma of Aorta.*—DR. HEWSON also exhibited a specimen of atheroma of the aorta, taken from a man, aged 44 years, a butcher, who died suddenly of an attack of gout affecting the heart. He had eaten a hearty supper of fish the evening before his death. The heart was much enlarged and gorged with blood, but contained no heart-clot; there was no pulmonary congestion.

*Fibroid Degeneration of Liver and Kidney; Spiculated Exostosis on Tibia and Fibula.*—DR. MORTON exhibited a specimen of spiculated exostosis on the tibia and fibula, from a patient whose leg was amputated on account of an indolent ulcer; also, the kidneys and liver from the same case, showing fibroid degeneration. The patient, Margaret Glenn, from whom the specimens were taken, was admitted into the Pennsylvania Hospital in January, 1858, on account of an indolent ulcer of the leg of thirty years' standing, which occupied nearly the entire circumference of the limb. She stated that at first it was small, but that repeated attacks of erysipelas left the ulcer larger and larger. Some of the veins of her leg were in a varicose condition; her health had been quite bad at times. She came into the Hospital for the purpose of having her limb amputated. She was about fifty-eight years of age, and in feeble health, with a good deal of gastric irritation, and irregularity of her bowels. Under tonics she improved much: her limb was removed by Dr. Peace. For some days after the operation her health and spirits seemed much better; but her appetite soon failed her, her bowels became quite loose, and she gradually sank, and died eleven days after the operation.

The tibia and fibula showed well the effects of a chronic ulcer, in producing a spiculated exostosis.

Autopsy.—All the organs were healthy with the exception of the kidneys and liver. The kidneys—the fibrous tunic was firmly attached to the organ, and when peeled off, brought some of the tissue with it; the kidneys were much lobulated, but the lobules quite small; on cutting the organ through, it gave the impression that it was fatty, from its striking resemblance to that degeneration, and was so pronounced, but its texture was more firm than is seen in that condition of the kidney. A microscopic examination showed an extensive fibrous deposit without any fat. The liver was very considerably increased in size, and presented also the fibrous deposit.

The attention of the Society was called to the occurrence of the fibroid deposit in the kidneys and liver, in connection with indolent ulcers. Gay ("on Indolent Ulcers") remarks that, "when a constitutional ulcer becomes indolent, there is much reason to apprehend some very serious lesion of an important organ, most frequently of the kidneys or of the liver." In the above case such was the condition.

DR. DARRACH, who had also examined a portion of the kidney with the microscope, stated that although the kidney presented the external appearance of oily degeneration, yet only a few oil globules were observed, but many fusiform cells; it was not easy to obtain a uriniferous tubule. When seen, they were perfectly or partially denuded of epithelium; their membrana propria seemed thickened; the liver presented a large excess of fibrous tissue. The case was interesting, as occurring in a person not addicted to drinking.

DR. WOODWARD alluded to the fact that enlargement of the bone, under chronic ulcers, had been noticed, and especially by Paget. The spiculæ in this case might have arisen as part of the hypertrophy, or from periostitis.

*Ossific Deposit projecting into Auricle.*—DR. FORBES exhibited a diseased heart, without any history, as it was obtained from the dissecting table. Its walls were thin, and its cavities enlarged. At the seat of the foramina of Thebesius were extensive ossific deposits, which projected into the right auricle.

*Aneurism of the Aorta bursting into a Bronchus.*—DR. FORBES next directed the attention of the Society to an aneurism which had ruptured into a bronchus. The patient had been attended by Dr. Turnbull, who gave the following account of the case:—I was called to see Nicholas Harris on the evening of the 20th March, 1858. He is a cutter for a large tailoring establishment in this city. He gave his age as thirty-two years; said he had been frequently sick, with pain and cough for more than a year; he had been a soldier during the Mexican war, when he contracted severe rheumatism, and had suffered since that time from intermittent fever. For the last six weeks he had been troubled with a severe influenza, and was much depressed on account of want of occupation; but a few days since he had been very much engaged cutting out clothing, causing him to stand and to stoop, and from the excessive fatigue he had fainted, and was brought home in a carriage. I found his skin warm and dry, his tongue coated and white, pulse feeble and rapid. He complained of pain in the upper part of the sternum, with a feeling of oppression.

Physical examination.—There was dulness on percussion over the whole of the left side of the chest, with bronchial respiration. On the right side percussion was morbidly clear, and auscultation discovered the inspiratory murmur quite feeble, with the expiratory murmur labored and wheezing. The action of the heart was rather loud. He complained of being feeble; could not sleep at night; would frequently get up on his hands and knees in bed to relieve his oppression. He had been taking a cough mixture made by his mother, which was exchanged for 10 grs. of Dover's powder, to be taken at bedtime.

On Saturday morning he felt better; had slept part of the night. Sunday morning, 9 o'clock, thirty-six hours after the first visit, he appeared languid; his skin was sallow; the pain in the front part of the chest had gone to the back. While half-reclining on a high settee, and resting on pillows, and speaking of his symptoms, he gave a deep moist cough, and raised his head to expectorate in a spittoon on the floor, and while in the act of turning, there came from his mouth and nose a series of gushes of blood, giving him no time to speak, or swallow, or breathe. He raised himself from the settee, and being unable to stand, the blood continuing to flow, he was placed on the floor on his side, when he gave a convulsive movement, and was found drowned in his own blood.

FIG. 5.

Post-mortem examination.–The body was natural, the heart normal; the aorta was much dilated at its arch, its coats thickened; an ulcer existed in them. The seat of perforation into the bronchial tube must have been small and then enlarged; the spot of perforation was eliptical, (see Fig. 5,) and partly closed by a new formation. The whole lung was filled with blood.

DR. LA ROCHE inquired into the state of the mucous membrane.

DR. FORBES stated that it was but little altered; a membrane seemed to stretch across the opening into the bronchial tube. With regard to the exact amount of dyspnœa latterly present, he did not know whether it had been a constant, or merely an occasionally violent symptom.

DR. STILLÉ remarked that the physical conditions were such, that, if any cause had disturbed the circulation in the part, difficulty of breathing by pressure on the membrane in the bronchial tube would have resulted.

*Fatty Liver.*—DR. HUMPHREYS exhibited a fatty liver, weighing nine and a half pounds, taken from a patient who died in the Pennsylvania Hospital, of effusion in the brain, consequent upon mania-a-potû. The kidneys were much enlarged.

*Kidneys and Brain of a Diabetic Person.*—DR. HUMPHREYS exhibited the kidneys and other organs of a case of diabetes. Pat. Dorken, aged forty-six, Irish, admitted into the Pennsylvania Hospital March 8th, with diabetes in an advanced stage. Had known of the existence of the disease for more than two years. He was much emaciated and exceedingly feeble, with great thirst, dry skin; passed daily from seven to ten pints of urine, of an average specific gravity of 1035. There were also well-marked symptoms of phthisis. About the 25th March a slight erysipelatous blush appeared upon the face and neck, accompanied with great prostration. The patient gradually sank, and expired March 28th.

Autopsy.—Right lung almost solidified, with small tortuous cavities at its top. Left lung healthy. Liver in a state of cirrhosis. Kidneys somewhat engorged, but otherwise healthy. Brain—membranes somewhat injected; dura mater somewhat adherent at the top.

*Colloid Cancer of the Omentum.*—DR. GROSS exhibited a specimen of colloid tumor, for which he expressed his obligations to Dr. Van Wyck, of this city, who had removed it a short time previously from a man, aged fifty-four years, a tailor by occupation, of spare make, and temperate habits. The morbid growth had been first observed about two years before death, having commenced in the left iliac region, from which it gradually extended in different directions until it filled the whole abdomen. Dr. Van Wyck had attended the man for about twelve months, when the tumor was nearly as large as at the post-mortem examination. The principal symptom was an extreme sense of weight and oppression in the abdomen, attended with considerable dyspnœa, especially when he suffered from constipation, to which he was very subject. He was but little emaciated, and there was hardly any œdema of the extremities. On dissection, it was found that the whole peritoneal cavity was occupied by an enormous colloid mass, reaching from the pelvis up to the diaphragm, which it had pushed high up into the chest, covering completely the abdominal viscera, and effacing all trace of the serous cavity. It weighed upward of twenty pounds, and was fully two inches and a half in thickness, whitish in its appearance, and composed of numerous characteristic cysts, from the volume of a mustard-seed to that of a small hickory-nut. All the abdominal, pelvic, and thoracic viscera were sound. A microscopic examination by Dr. Da Costa, showed small cells, indistinctly nucleated, fibres, and an amorphous basement substance.

DR. GROSS stated that he himself had seen two similar examples, which are briefly described in the new edition of his *Pathological Anatomy.* The subject of the first case had been a man, forty-nine years old, who had an enormous colloid tumor, which extended from the liver to the pelvis, and involved the colon and greater part of the stomach, the abdominal viscera being greatly compressed and almost hidden behind the morbid mass. In weight it was found to be about twenty-five pounds, in thickness from two and a half to three inches, and measured in length nearly one foot, and in breadth over eight inches. In structure it was made up of innumerable masses, varying in size from a mustard-seed to a hickory-nut, and its appearances were denotive of its having been developed in the peritoneum or great omentum, the latter of which was effaced. The liver was diminished in volume, from the pressure of the tumor, but was otherwise healthy, and the thoracic and abdominal viscera were perfectly normal.

The second case occurred in a gentleman, forty-five years of age, who died in a state of complete exhaustion, after the disease had existed for about two years. The whole peritoneal cavity, with the exception of a small portion over the right lobe of the liver, was obliterated and occupied by an immense tumor, extending from the neck of the bladder to the diaphragm, which was pushed high up in the chest. All the pelvic and abdominal viscera were inclosed in the mass, but were free from colloid disease. The colloid structure was well marked, being composed of cells, the largest of which were about the size of a hazel-nut, and filled with a pale greenish jelly-like substance.

DR. HEWSON inquired if dropsy had been present.

DR. GROSS. None.

DR. HEWSON had had three cases of colloid of the peritoneum come under his notice. Two occurred in one family. Both had marked dropsy, and died within one year of each other. In both cases the surface of the peritoneum was studded with colloid. In a third case, the colloid deposit was most marked on the under surface of the liver, and on the surface of the uterus and ovaries. A large cyst in the ovary was filled with colloid. This case was tapped during life, and a serous fluid evacuated, which gelatinized on standing.

## Figures and Tables

**Fig. 5. f1:**